# Accuracy of infusion flow rates and bolus doses for portable infusion pump

**DOI:** 10.1038/s41598-025-98533-8

**Published:** 2025-04-19

**Authors:** Ryo Sekiguchi, Michiko Kinoshita, Yuki Maeda, Katsuya Tanaka

**Affiliations:** 1 Department of Anesthesiology, Tokushima University Hospital, Tokushima, 2-50-1 Kuramoto-Cho, Tokushima-Shi, Tokushima 770-8503 Japan; 2 Department of Anesthesiology, Institute of Biomedical Sciences, 3-8-15, Tokushima, 770-8503 Japan

**Keywords:** Accuracy, Elastomeric balloon pump, Electronic pump, Portable disposable infusion pump, Regional analgesia, Pain, Drug delivery, Pain management

## Abstract

This study aimed to investigate the flow rate accuracy and bolus doses of four portable pumps with different mechanisms. We evaluated two electronic (COOPDECH Amy PCA [Amy] and CADD-Solis) and two elastomeric balloon-type pumps (COOPDECH Balloonjector and Rakurakufuser) under the following conditions: placement with an epidural catheter, operation on a shaking table, warming to 32 °C, and at room temperature (control, 25 °C). Amy maintained consistent flow rates across all conditions. The CADD-Solis also exhibited consistent flow rates, with minor yet statistically significant changes upon epidural catheter placement (− 5.0%, P = 0.005) and motion conditions (4.2%, P = 0.015). The Balloonjector and Rakurakufuser exhibited flow rate variations over time, and temperature increases significantly increased flow rates by 24.3% (P < 0.001) and 20.3% (P < 0.001), respectively. Bolus volume accuracy for the Amy, Balloonjector, and Rakurakufuser was not significantly affected under different conditions. The CADD-Solis showed a slight decrease in bolus volume with an epidural catheter (mean difference, 0.3 mL; P = 0.003). The electronic pumps maintained consistent flow rates across various conditions, whereas elastomeric balloon pumps exhibited variable rates influenced by time and temperature, increasing the risk of medication overdose. Bolus dosing accuracy was clinically satisfactory for all pump mechanisms.

## Introduction

Portable infusion pumps are compact, versatile devices used to deliver a range of medication, including local anesthetics and opioids for analgesia^1,2^, chemotherapeutic agents^3,4^, and antimicrobial therapies^5–8^. These pumps support ambulatory care and reduce healthcare costs^9,10^. A previous North American study reported that approximately 85% of the anesthesiologists affiliated with the American Society of Regional Anesthesia and Pain Medicine used these pumps for home catheter infusion, providing continuous peripheral nerve blocks for postoperative analgesia^1^. Given the possible lack of continuous medical supervision, the reliability of these pumps is crucial for ensuring consistent therapeutic effectiveness and side effect management^5,11^.

Commonly utilized mechanisms in portable infusion pumps are elastomeric balloons and electronic types^1^. Prior research has shown a patient preference for the elastomeric balloon type because of its simplicity, light weight, and user-friendliness^3,12^. However, factors, such as changes in balloon-reservoir size over time^13,14^, temperature^13,14^, outlet resistance^15–17^, atmospheric pressure^18^, and the pump’s height relative to the outlet site^17,19^, can influence the infusion flow rates of the elastomeric pumps. By contrast, electronic pumps offer more precise and flexible adjustable dosing regimens^2^. A recent advancement in this field involves an electronic portable pump featuring disposable, ultrasmall micropump technology measuring 10 mm × 10 mm × 2 mm. This miniaturization is expected to improve portability by reducing weight while maintaining accuracy^20^. However, the exactitude of electronic pumps equipped with micropump technology has not yet been thoroughly investigated. Additionally, the stability of the infusion flow rates under conditions that assume a patient’s dynamic activity and the accuracy of bolus dosing have not been assessed for either pump type.

This study aimed to address three knowledge gaps: accuracy of electronic pumps equipped with micropump technology; continuous dosing accuracy in dynamic motion; accuracy of bolus dosing. This study examined four commercially available pumps (one electronic featuring micropump technology, one standard electronic, and two elastomeric balloons) and assessed their performance in terms of continuous dosing accuracy under temperature fluctuations, during epidural catheter placement, in dynamic motion conditions, and for bolus dosing accuracy.

## Methods

**Fig. 1:**
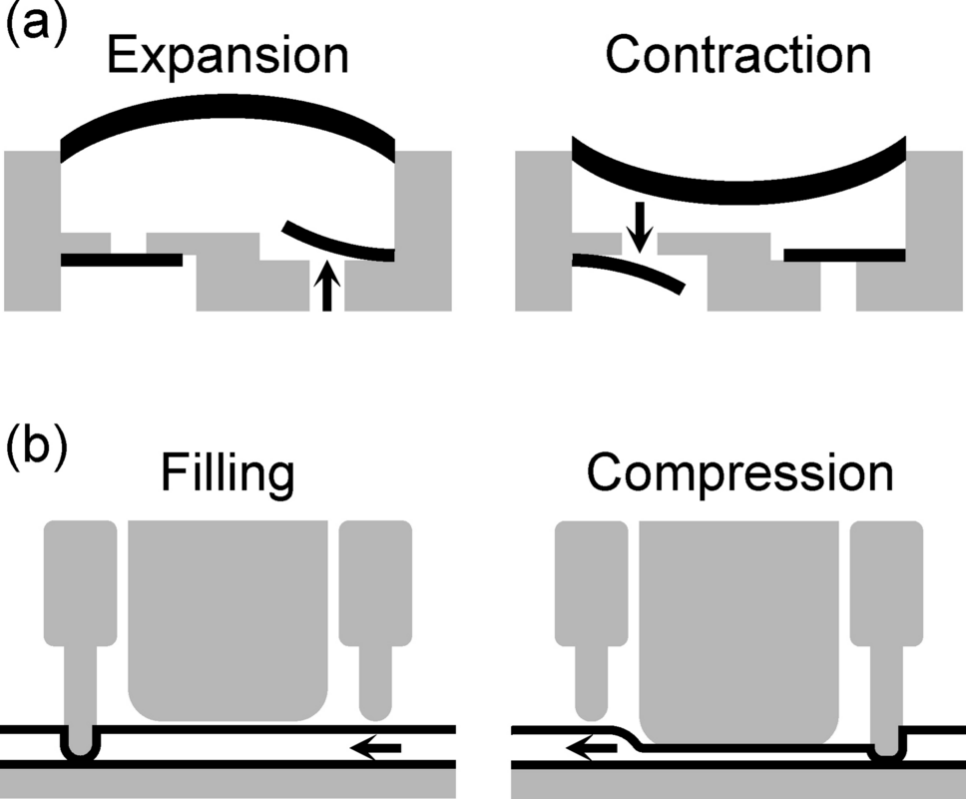
Fluid delivery systems of electronic pumps: (**a**) COOPDECH Amy PCA: The micropump measures 10 mm × 10 mm × 2 mm. During pump expansion, the upstream valve opens to draw fluid in, while during contraction, the downstream valve opens to discharge the solution. (**b**) CADD-Solis: The device employs three pistons that sequentially compress an infusion tube. Coordinated movement of upstream, middle, and downstream pistons alternates between occlusion and release to drive fluid delivery through peristaltic action.

This study did not require approval from an institutional ethics board. In this study, four types of commercially available portable pumps were used (Table 1, Supplemental Table S1). The COOPDECH Amy PCA (Daiken Medical Co., Ltd., Osaka, Japan) is the electronic type, featuring ultrasmall micropump technology; CADD-Solis (Smiths Medical Japan Ltd., Tokyo, Japan) served as the standard electronic type; COOPDECH Balloonjector (Daiken Medical Co., Ltd.) and Rakurakufuser (Smiths Medical Japan Ltd., equivalent to VESSEL FUSER; AuBEX Corp., Tokyo, Japan) were the elastomeric balloon type. Different fluid delivery systems of two electronic pumps are illustrated in Fig. 1. These pumps were equipped with an adjustable flow rate system and bolus delivery functionality. All devices complied with Japanese standards. The two elastomeric balloon pumps complied with the standards for pressurized medicinal infusion pumps approved by the Ministry of Health, Labour and Welfare. These standards require a flow rate accuracy within ± 20% of the nominal rate when tested with either saline or a 5% glucose solution at body surface temperature (32 °C ± 2 °C) or room temperature (23 °C ± 2 °C), depending on the intended use conditions. The two electronic types conformed to JIS (Japanese Industrial Standards) T0601-2–24, which is equivalent to the International Electrotechnical Commission (IEC) 60,601–2–24.

**Table 1:** Profiles of the portable infusion pumps evaluated in this study.

Pump	Reservoir volume (mL)	Adjustable flow rate (mL/h)	Power source	Calibration temperature (°C)	Flow rate accuracy*	Weight (g)
COOPDECH Amy PCA	100	0.1–30.0	Electronic (micropump)	25	± 6%	140
CADD-Solis	100	0–100	Electronic (squeezing)	2–40	± 6%	595
COOPDECH Balloonjector	200	2, 4, 6	Elastomeric (balloon)	23	± 10%	153
Rakurakufuser	150	1–7 (1 mL increments)	Elastomeric (balloon)	30	± 10%	135

* Manufacturer specifications.

**Fig. 2:**
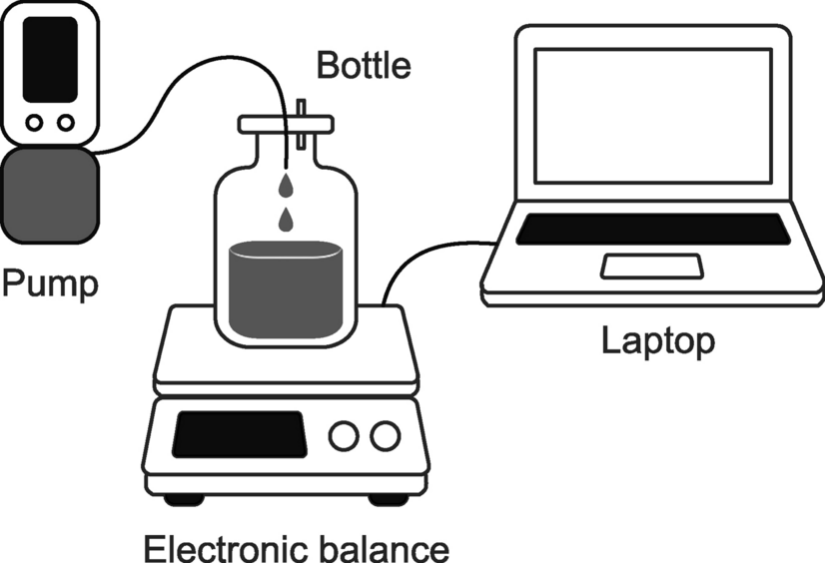
System used for measuring the flow rates of portable infusion pumps.

The pumps were filled with 0.9% normal saline to their maximum capacity and set at an infusion flow rate of 4 mL/h. Fluid was collected in a glass bottle placed on a recently calibrated electronic balance (A&D GX 6002 A [weighing capacity = 6200 g, scale division d = 0.01 g, verification scale interval e = 0.1 g], A&D Company, Tokyo, Japan) connected to a laptop (Fig. 2). The use of glass bottles eliminated electrostatic interference, and the total weight of the filled container fell within the manufacturer’s recommended optimal weighing range. The top of the collection bottle was sealed with plastic wrap to minimize evaporation. Weight measurements were recorded every 10 min, and hourly flow rates were calculated based on these readings. This gravimetric method of measuring flow rates was adopted based on previous similar studies^13–19^. The dosing period was considered complete when the measured flow rate decreased to < 2.0 mL/h (50% of the set infusion flow rate). The room temperature was maintained at 25 °C, and the height of the pumps relative to the outlet site was kept constant. Disposable devices were used only once; the COOPDECH Amy PCA was recharged before each experiment, and the CADD-Solis was equipped with new batteries for each experiment.

The experiments were conducted under four conditions: (1) epidural catheter condition, measurements with an epidural catheter (Perifix ONE Catheter, 20 gauge, 100 cm; B. Braun, Melsungen, Germany); (2) motion condition, measurements conducted while placing pumps on a shaking table (Mild Mixer PR-36; Taitec Corporation, Saitama, Japan; setting: 50% speed, low angle); (3) warming condition, measurements with pumps warmed in an incubator set at 32 °C (Sanyo Incubator MIR-153; Sanyo, Osaka, Japan); (4) control condition, baseline measurements without any additional manipulation. The bolus doses (3 mL in each pump) were measured under the same four conditions as previously mentioned.

The accuracy of the continuous infusion flow rate was evaluated using two criteria: the average measured flow rate as a percentage of the set flow rate (4 mL/h) during the dosing period (%); the percentage of dosing time within the range of 100% ± 15% of the set flow rate during the dosing period (%). The criterion of within 100% ± 15% was based on benchmarks established by similar studies^13,14,19,21^. The accuracy of bolus dosing was assessed based on the measured bolus dose volumes (in milliliters).

All experiments were performed in triplicates, and considering precedents from similar studies, three measurements were deemed sufficient^13,14,19,21^. Results are expressed as mean (standard deviation). Comparisons among conditions were performed using a one-way analysis of variance (ANOVA). When the ANOVA results were significant, Tukey’s post-hoc test was used for comparison with the control condition. Statistical significance was set at P < 0.05. All statistical analyses were performed using EZR (Saitama Medical Center, Jichi Medical University, Saitama, Japan), which is a graphical user interface for R (The R Foundation for Statistical Computing, Vienna, Austria). Specifically, it is a modified version of the R commander designed to incorporate statistical functions frequently used in biostatistics^22^.

## Results

**Fig. 3:**
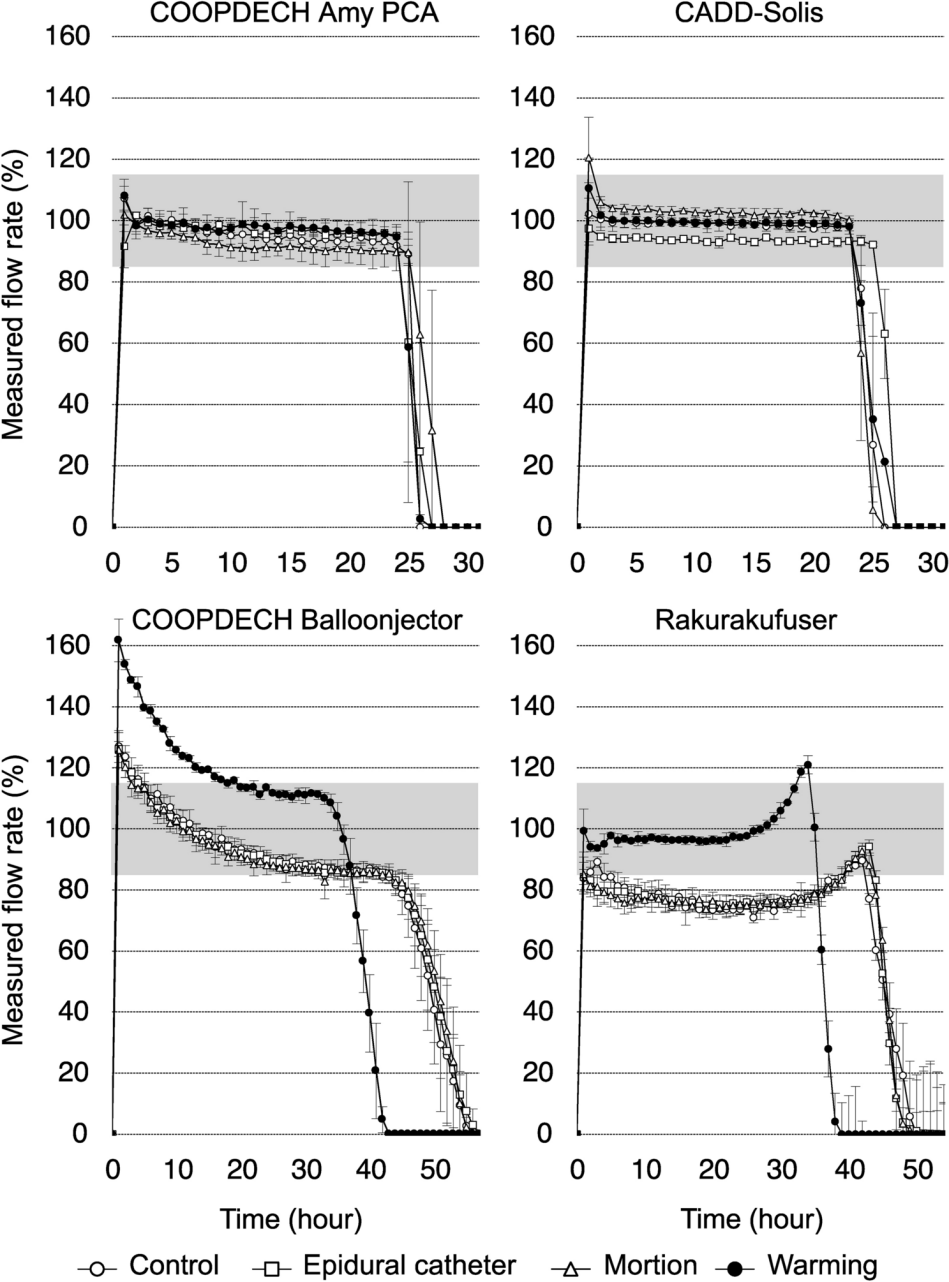
Measured flow rate profiles during the dosing period. The data illustrate the actual measured infusion flow rate as a percentage of the set flow rate (4 mL/h) (%). Control, corresponds to measurements at room temperature (25 °C); epidural catheter, indicates the use of a 20-gauge, 100-cm epidural catheter; motion, represents the condition on a shaking table; warming, denotes warming to 32 °C; gray zone, 100% ± 15% range of the set flow rate. The axis labels are consistent across all panels. Data are presented as mean (standard deviation).

The measured flow rate profiles during the dosing period are shown in Fig. 3. Under control conditions, both COOPDECH Amy PCA and CADD-Solis exhibited consistent flow rates throughout the dosing period. However, the COOPDECH Balloonjector showed a gradual decline in flow rate from the start to the end of administration, whereas Rakurakufuser displayed an accelerated flow rate in the latter half before cessation.

Table 2 presents the average measured flow rate as a percentage of the set flow rate (4 mL/h) during the dosing period for each condition. The COOPDECH Amy PCA showed no significant changes in flow rates across conditions. The CADD-Solis showed a slightly reduced flow rate with the epidural catheter [93.1% (0.5%); mean difference, − 5.0%; 95% confidence interval (CI), − 1.7 to − 8.3 l; P = 0.005] and a marginally increased flow rate during motion [102.4% (0.7%); mean difference, 4.2%; 95% CI, 0.9 to 7.6; P = 0.015], both statistically significant yet small compared with the control condition [98.1% (1.6%)]. The COOPDECH Balloonjector showed a significant increase in the flow rate during warming [117.5% (0.7%); mean difference, 24.3%; 95% CI, 19.1 to 29.6; P < 0.001] compared with the control condition [98.2% (2.5%)]. Similarly, the Rakurakufuser significantly increased the flow rate during warming [99.3% (3.9%); mean difference, 20.3%; 95% CI, 11.6 to 29.1; P < 0.001] compared with the control condition [78.9% (3.6%)].

**Table 2:** Average measured flow rate as a percentage of the set infusion rate (4 mL/h) during the dosing period (%).

Pump	Control	Epidural catheter	Motion	Warming	P value
COOPDECH Amy PCA	95.2 (1.9)	96.0 (2.7)	91.8 (3.8)	97.0 (3.2)	0.231
CADD-Solis	98.1 (1.6)	93.1 (0.5)*^1^	102.4 (0.7)*^2^	99.3 (1.7)	< 0.001
COOPDECH Balloonjector	98.2 (2.5)	92.3 (3.0)	91.4 (1.0)	117.5 (0.7)*^3^	< 0.001
Rakurakufuser	78.9 (3.6)	78.8 (2.3)	78.5 (3.3)	99.3 (3.9)*^4^	< 0.001

Data are presented as mean (standard deviation).

The P values were derived using analysis of variance. P values for the post-hoc tests: *^1^vs. control, P = 0.005; *^2^vs. control, P = 0.015; *^3^vs. control, P < 0.001; *^4^vs. control, P < 0.001.

Table 3 presents the percentage of dosing time within the range of 100% ± 15% of the set flow rate during the dosing period. Both the COOPDECH Amy PCA and CADD-Solis showed no significant changes in accuracy within the 100% ± 15% range across conditions. Meanwhile, the COOPDECH Balloonjector showed significant differences across conditions, post-hoc analysis revealed no specific condition with a significant change compared with the control. In the warming condition, the Rakurakufuser exhibited a significantly higher percentage of dosing time within the 100% ± 15% accuracy range [89.1% (0.5%); mean difference, 58.2%; 95% CI, 31.8 to 84.5; P < 0.001] relative to the control [31.0 (12.2%)].

**Table 3:** Percentage of dosing time within 100% ± 15% of set flow rate (4 mL/h) during the dosing period (%).

Pump	Control	Epidural catheter	Motion	Warming	P value
COOPDECH Amy PCA	93.2 (8.4)	88.1 (17.2)	89.6 (13.6)	94.8 (2.8)	0.887
CADD-Solis	96.3 (1.4)	94.0 (1.3)	95.1 (1.3)	93.4 (4.3)	0.521
COOPDECH Balloonjector	65.5 (8.9)	68.3 (10.5)	66.1 (10.1)	43.3 (4.7)	0.027
Rakurakufuser	31.0 (12.2)	17.4 (5.8)	18.8 (14.9)	89.1 (0.5) *	< 0.001

Data are presented as mean (standard deviation).

The P values were derived using analysis of variance. P value for the post-hoc test: *vs. control, P < 0.001.

Table 4 presents the measured volumes of the bolus doses (3 mL). For the COOPDECH Amy PCA, COOPDECH Balloonjector, and Rakurakufuser, there were no significant changes in bolus volumes across different conditions. The CADD-Solis showed a statistically significant decrease in bolus volumes when used with an epidural catheter [2.7 (0.1) mL] compared with the control condition [3.0 (0.1) mL]; however, the mean difference was minimal (− 0.3 mL; 95% CI, − 0.1 to − 0.5; P = 0.003).

**Table 4:** Measured volumes of bolus doses (3 mL).

Pump	Control	Epidural catheter	Motion	Warming	P value
COOPDECH Amy PCA	3.0 (0.0)	3.0 (0.1)	3.1 (0.1)	3.0 (0.0)	0.499
CADD-Solis	3.0 (0.1)	2.7 (0.1)*	3.1 (0.0)	3.1 (0.1)	< 0.001
COOPDECH Balloonjector	3.0 (0.0)	3.0 (0.1)	2.9 (0.1)	3.0 (0.1)	0.465
Rakurakufuser	3.0 (0.1)	3.0 (0.1)	3.1 (0.1)	3.1 (0.2)	0.445

Data are presented as mean (standard deviation).

The P values were derived using analysis of variance. P value for the post-hoc test: *vs. control, P = 0.003.

## Discussion

In this study, we assessed the infusion flow rate accuracy of four pumps under four different conditions: control, during epidural catheter placement, in dynamic motion, and with warming. The COOPDECH Amy PCA, an electronic pump featuring novel micropump technology, maintained consistent flow rates across all conditions. The standard electronic CADD-Solis also showed consistent flow rates, with slight changes (approximately 5%) observed during epidural catheter placement and motion conditions. In contrast, the elastomeric pumps, COOPDECH Balloonjector and Rakurakufuser, demonstrated dynamic changes in flow rates over time. Additionally, an increase in temperature from 25 °C to 32 °C significantly increased the flow rates of both elastomeric pumps by approximately 20%.

The two electronic pumps evaluated in this study use different mechanisms (as shown in Fig. 1); the COOPDECH Amy PCA uses an ultrasmall micropump that cyclically expands and contracts for infusion delivery, whereas the CADD-Solis administers by sequentially compressing (squeezing) the infusion tubes. Both pumps consistently maintained flow rates within the 100% ± 15% range under warming conditions or with the resistance of an epidural catheter. Stable flow has been highlighted as an asset for electronic pumps in previous studies^13,14,16,17^, and our results further indicate that this stability may not depend on their operating mechanisms. Moreover, this is the first study to demonstrate the stability of electronic pumps during dynamic motion. Although previous studies have indicated that the flow rates of electronic pumps can be affected by battery depletion or replacement, and component failures^14,19,23^, such scenarios were not explored in our research.

This study showed that the flow rates of the elastomeric balloon pumps exhibited significant changes over time and with temperature variation, consistent with the findings of previous studies^13,14,19,21,24^. The flow rates of these elastomeric pumps adhere to the Hagen-Poiseuille law, which states that the flow rate is directly proportional to the fourth power of the catheter radius and infusion pressure and inversely proportional to the fluid viscosity and catheter length^15^. The extent of balloon contraction affects infusion pressure, whereas temperature alters fluid viscosity. Despite a previous study suggesting an influence on flow rates with microcatheters as narrow as 28 gauge^15^, our results showed no such effect with a 20-gauge epidural catheter on elastomeric pump flow rates. Because of potential catheter rupture, the use of catheters thinner than 24 gauge is not currently recommended^15,25^.

We evaluated the accuracy of bolus dosing, a novel contribution of this study. Our findings demonstrate that electronic pumps consistently achieved high accuracy in both flow rates and bolus dosing, reinforcing their reliability for clinical use. Numerous studies have established that a regimen incorporating scheduled intermittent or patient-controlled bolus dosing outperforms continuous infusion alone in regional anesthesia for pain management^26–29^. This underscores a notable clinical demand for accurate bolus dosing, and the insights from our study on its precision may hold clinical significance.

In contrast, elastomeric pumps demonstrated significant instability in flow rates and sensitivity to temperature changes, raising serious patient safety concerns. Notably, at 32 °C, the COOPDECH Balloonjector delivered an initial flow rate approximately 160% higher than expected, while at 25 °C, the Rakurakufuser consistently delivered less than 80% of the expected rate. These variations are particularly concerning in ambulatory settings, where environmental temperature fluctuations are often uncontrolled, and deviations in flow rates can go undetected. Elevated flow rates during postoperative pain management increase the risk of opioid-related complications, such as respiratory depression or nausea^30^, and local anesthetic systemic toxicity^31^. Conversely, reduced flow rates can result in inadequate pain control.

Our findings highlight electronic pumps as the more reliable option for consistent drug delivery. However, in facilities limited to elastomeric pumps, we recommend a risk mitigation strategy of using patient-controlled analgesia (PCA) with bolus dosing instead of continuous infusion, as bolus doses showed consistent accuracy across varying conditions.

It is important to interpret these in vitro results cautiously when applying them to clinical settings, as various in vivo factors can affect delivery accuracy. Previous studies have demonstrated that back pressure and vertical displacement of pumps can significantly influence delivery accuracy^17,32^. Factors such as physiological venous pressure and changes in pump positioning relative to the infusion site should also be considered in clinical practice. Additionally, prior research reported that 20.5% of elastomeric balloon pumps failed to deflate correctly in clinical settings^33^. The absence of alarm systems in these pumps complicates the detection of malfunctions, though our study did not specifically address these issues.

This study had some limitations. First, the evaluated pumps had different manufacturer-recommended temperatures (listed in Table 1). The room temperature was maintained at 25 °C during experiments, which may not have been ideal for some pumps. Owing to this variation, direct comparisons between different pumps are difficult. Second, the pumps had different reservoir volumes, which could have influenced the dosing duration and flow rate accuracy assessment. Nonetheless, the pumps were filled to their maximum capacity, as recommended by the manufacturer, to achieve optimal performance. Third, an accuracy assessment was performed at a flow rate of 4 mL/h. Although this is a clinically relevant setting, different flow rates may produce different results because the pumps have adjustable flow rate systems (Table 1). Fourth, we used 0.9% normal saline for the experiments, consistent with previous studies^13–15,17,19^. However, as fluid viscosity varies with depending on the type and concentration, the results may not fully apply to other fluids^34^. The viscosity of 0.9% normal saline was assumed to be comparable to that of 0.5% ropivacaine^13^. For different fluids, measuring viscosity would be valuable to predict flow rate accuracy and enhance patient safety. Fifth, we did not evaluate variability between different manufacturing batches or the effects of shelf-life. Previous studies have shown that storage conditions, such as refrigeration or freezing, can influence flow stability^35,36^. Additionally, the devices used in this study were approved under Japanese regulatory standards. While the electronic pumps met JIS T0601-2–24 (equivalent to international IEC 60,601–2–24), the compliance of elastomeric pumps with international standards like ISO 28,620 could not be confirmed, which may limit the global applicability of our findings.

In conclusion, the electronic pumps COOPDECH Amy PCA and CADD-Solis maintained flow rates despite changes in conditions, such as epidural catheter placement, dynamic motion, and warming. The elastomeric balloon pumps, COOPDECH Balloonjector and Rakurakufuser, showed flow rate variability over time and with temperature fluctuations, posing a potential risk of unintended medication overdose. The accuracy of bolus dosing was clinically satisfactory, showing minimal impact of various conditions, regardless of the pump mechanism. These findings underscore the importance of selecting the appropriate pump type based on specific clinical scenarios to ensure optimal infusion regimens.

## Supplementary Information

Supplementary information

## Data Availability

The datasets used and/or analyzed during the study are available from the corresponding author on reasonable request.
