# Supplementary material for: Accuracy of infusion flow rates and bolus doses for portable infusion pump
Source: Sci Rep. 2025 Apr 19;15:13517. doi: 10.1038/s41598-025-98533-8 (PMC12009376; doi:10.1038/s41598-025-98533-8)
Supplement: Supplementary file 1 — Supplementary information [file 41598_2025_98533_MOESM1_ESM.docx]

**Supplemental Table S1.** Profiles of the portable infusion pumps evaluated in this study.

| Pump | Manufacturer | Product number | Website* |
| --- | --- | --- | --- |
| COOPDECH Amy PCA | Daiken Medical Co. | CAP-100 | https://daiken-iki.co.jp/en/pi/seihin_amy.html |
| CADD-Solis | Smiths Medical Japan Ltd. | 21-2111-0300-09 | https://www.icumed.com/products/infusion-therapy/infusion-pumps-and-software/cadd-pain-and-ambulatory-pumps/cadd-solis-infusion-system/ |
| COOPDECH Balloonjector | Daiken Medical Co. | CIE20N-2040-IP3-E3020 | https://daiken-iki.co.jp/en/pi/seihin_be.html |
| Rakurakufuser | Smiths Medical Japan Ltd. | 21-ABX-N300P3F7 | https://www.aubex.co.jp/en/product/medical/ |

* Accessed on November 15, 2024.
